# Supplementary material for: Biological effects of the loss of homochirality in a multicellular organism
Source: Nat Commun. 2022 Nov 18;13:7059. doi: 10.1038/s41467-022-34516-x (PMC9674851; doi:10.1038/s41467-022-34516-x)
Supplement: Supplementary file 1 — Supplementary Information [file 41467_2022_34516_MOESM1_ESM.pdf]

Supplementary Information

Title: Biological effects of the loss of homochirality in a multicellular organism

Authors: Agnes Banreti[1]*, Shayon Bhattacharya[2]†, Frank Wien[3]†, Koichi Matsuo[4], Matthieu Réfrégiers[5], Cornelia Meinert[6], Uwe Meierhenrich[6], Bruno Hudry[1]††, Damien Thompson[2]††, Stéphane Noselli[1]

Affiliations: [1]Université Côte d'Azur, CNRS, Inserm, Institut de Biologie Valrose; 06108, Nice, France.

[2]Department of Physics, Bernal Institute, University of Limerick; V94 T9PX, Limerick, Ireland.

[3]DISCO Beamline, Synchrotron SOLEIL; 91192, Gif-sur-Yvette, France.

[4]HiSOR Hiroshima Synchrotron Radiation Center, Hiroshima University; Hiroshima, Japan.

[5]Centre de Biophysique Moléculaire, CNRS; UPR4301, 45071 Orléans, France.

[6]Université Côte d'Azur, Institut de Chimie de Nice, CNRS; UMR 7272, 06108 Nice, France.

† , †† These authors contributed equally to this work.

*Correspondence: Agnes.Banreti@univ-cotedazur.fr

Supplementary Figures

Supplementary Figure legends

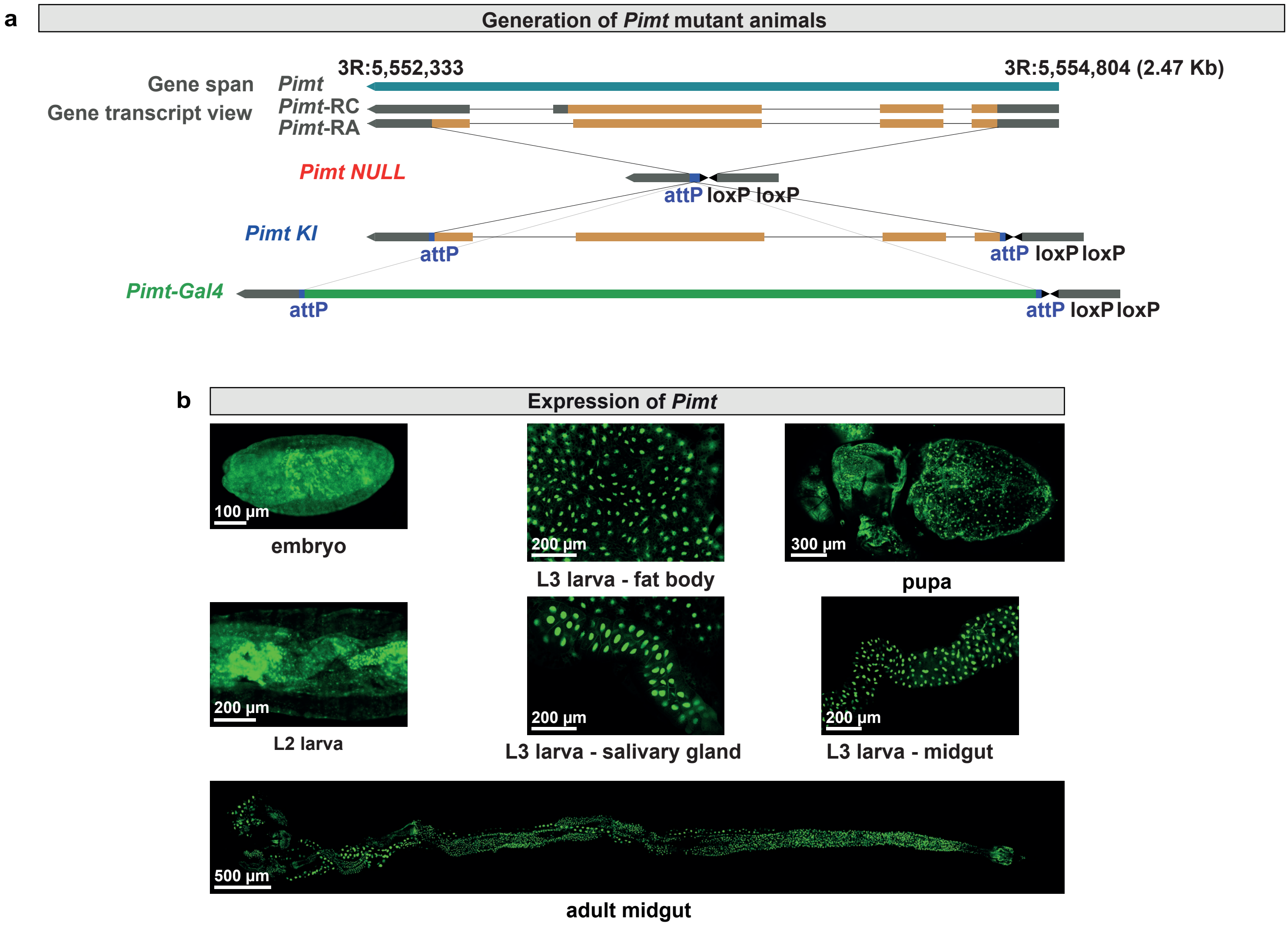
a
Generation of Pimt mutant animals
3R:5,552,333
3R:5,554,804 (2.47 Kb)
Gene span
Pimt
Gene transcript view
Pimt-RC
Pimt-RA
Pimt NULL
attP loxP loxP
Pimt KI
attP
attP loxP loxP
Pimt-Gal4
attP
attP loxP loxP
b
Expression of Pimt
100 µm
embryo
200 µm
L3 larva - fat body
300 µm
pupa
200 µm
L2 larva
200 µm
L3 larva - salivary gland
200 µm
L3 larva - midgut
500 µm
adult midgut

Supplementary Figure 1: *Pimt* is evolutionary conserved and constitutively expressed in *Drosophila*

a, The *Pimt* locus and strategy for the generation of *Pimt* null mutant allele, *Pimt*^{n1} (*Pimt NULL*), *Pimt* knock-in (*Pimt KI*) and *Pimt-Gal4* lines. Shown are coding exons (orange), 5' and 3' untranslated regions (UTR, grey) and introns (black lines). For detailed description of the cloning strategies, see Methods.

b, Spatio-temporal expression of Pimt using *Pimt-Gal4* and *UAS-Stinger*^{NLS}. Whole embryo stained with anti-GFP**;** L2: Stage 2 larva; fat body-, salivary gland-, and midgut of stage 3 larvae (L3); whole stage 9 pupa and adult midgut. Images show PFA fixed and immunostained (E), or unfixed live specimens (L2, L3, E and AMG). Scale bars correspond to 100 μm (E), 200 μm (L2,L3), 300 μm (P) and 500 μm (AMG). Each experiment was repeated independently at least three times.

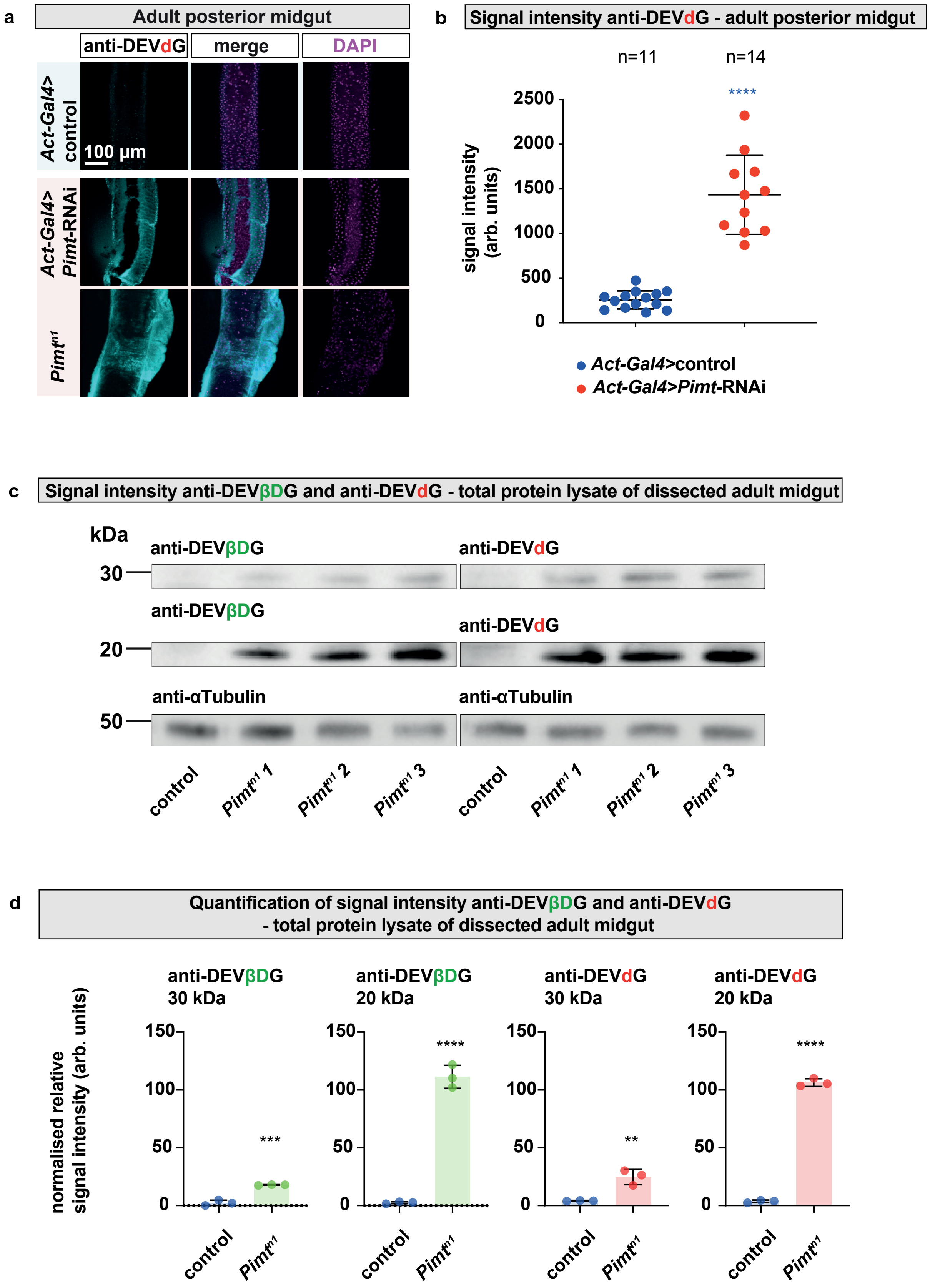
a
Adult posterior midgut
anti-DEVdG
merge
DAPI
Act-Gal4> control
100 µm
Act-Gal4> Pimt-RNAi
$Pimt^{n1}$
b
Signal intensity anti-DEVdG - adult posterior midgut
n=11
n=14
****
signal intensity (arb. units)
2500
2000
1500
1000
500
0
Act-Gal4>control
Act-Gal4>Pimt-RNAi
c
Signal intensity anti-DEVβDG and anti-DEVdG - total protein lysate of dissected adult midgut
kDa
anti-DEVβDG
anti-DEVdG
30
anti-DEVβDG
anti-DEVdG
20
anti-αTubulin
anti-αTubulin
50
control
$Pimt^{n1}$ 1
$Pimt^{n1}$ 2
$Pimt^{n1}$ 3
control
$Pimt^{n1}$ 1
$Pimt^{n1}$ 2
$Pimt^{n1}$ 3
d
Quantification of signal intensity anti-DEVβDG and anti-DEVdG - total protein lysate of dissected adult midgut
anti-DEVβDG 30 kDa
anti-DEVβDG 20 kDa
anti-DEVdG 30 kDa
anti-DEVdG 20 kDa
normalised relative signal intensity (arb. units)
150
100
50
0
***
****
**
****
control
$Pimt^{n1}$

Supplementary Figure 2: The pathophysiological outcomes of Pimt loss of function in heterochiral animals

a, Dissected adult female gut stained with anti-DEV***d***G antibody. Guts of control animals do not show any immunoreactivity, while *Act-Gal4>Pimt*-RNAi and $Pimt^{n1}$ show positive staining for the DEV***d***G-specific antibody.

b, Quantification of signal intensities of the DEV***d***G-specific immunostainings shown in (a). Each experiment was repeated independently at least three times, n=number of animals. *P*-value from two-sided Mann-Whitney U-test is ****p<0.0001.

c, Western blot analysis of lysates of dissected adult guts from control and $Pimt^{n1}$ chiral-deficient animals (shown are technical and biological triplicates) with the anti-DEV***d***G and anti-DEV**iso*D***G antibodies.

d, Quantification of c,. Signal intensities were normalised to anti-α-Tubulin control. Each experiment was repeated independently at least three times. For b, and c, values are presented as average ± standard deviation (S.D.). P values from unpaired, two-tailed Student's t-tests are ****p<0.0001; ***p=0.0004; **p=0.0056. All samples derive from the same experiment and gels/blots were processed in parallel for c, and d,. Source data are provided as Source Data file for b, and d,.

Characterisation of chiral amino acids, homochiral and heterochiral peptides

a Structure of D-Asp and L-Asp

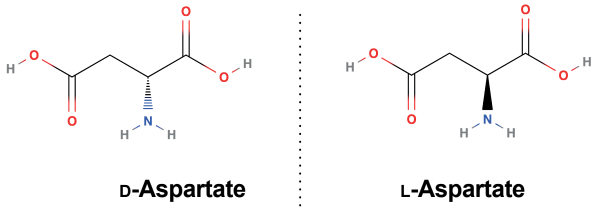

b SRCD of D-Asp and L-Asp films

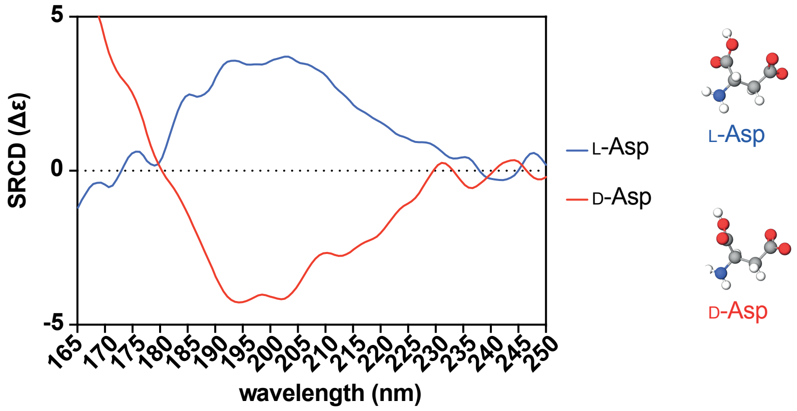

c SRCD of peptides

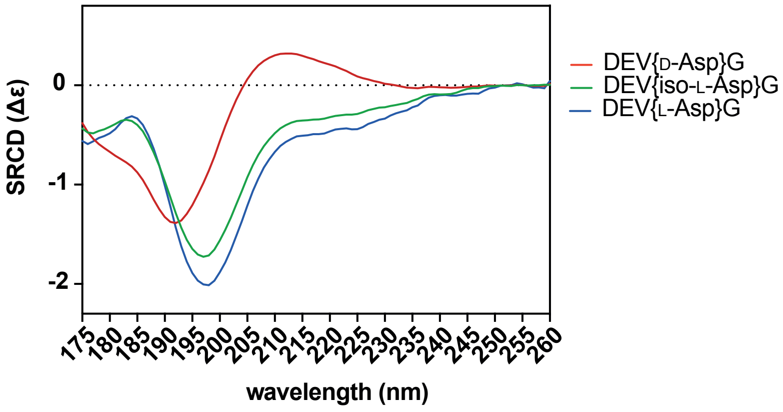

Supplementary Figure 3: Far-UV synchrotron radiation circular dichroism (SRCD) spectra of synthetic oligopeptides

a, Chemical structures and stick representation of L-aspartate (L-Asp) and D-aspartate (D-Asp). Note the Asp sidechain becomes deprotonated to form a negatively charged COO^- carboxylate group at physiological pH.

b, Far-UV SRCD spectra of 68 Å thick amorphous films of L-aspartate (L-Asp) and D-aspartate (D-Asp).

c, Recorded far-UV SRCD spectra of synthetic oligopeptides with mixed chirality (peptides, incorporating one iso-L- or D-aspartic acid residue). Source data are provided as a Source Data file for b, and c,.

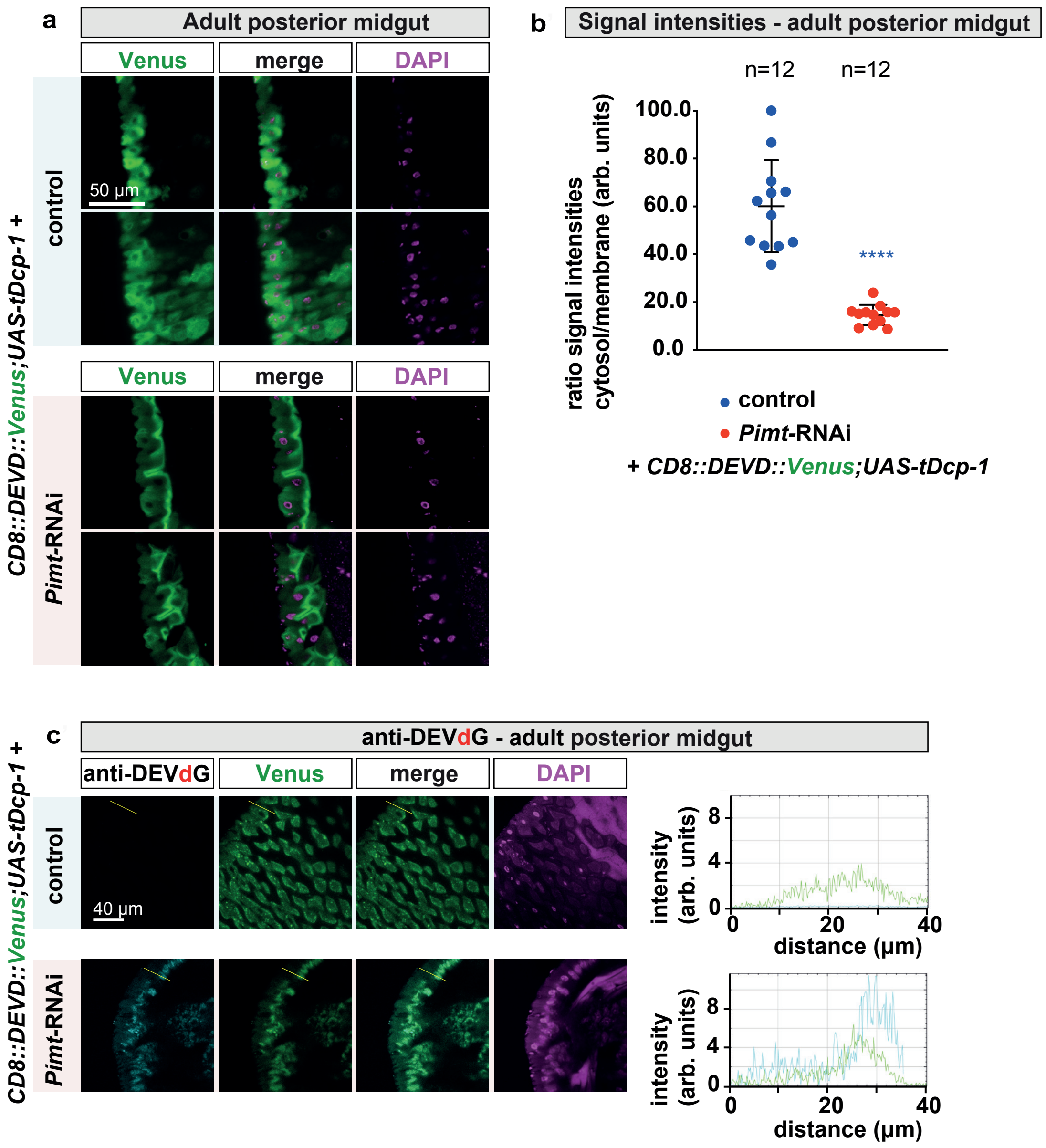
a
Adult posterior midgut
Venus
merge
DAPI
control
50 µm
Pimt-RNAi
CD8::DEVD::Venus;UAS-tDcp-1 +
b
Signal intensities - adult posterior midgut
n=12
n=12
ratio signal intensities cytosol/membrane (arb. units)
100.0
80.0
60.0
40.0
20.0
0.0
****
control
Pimt-RNAi
+ CD8::DEVD::Venus;UAS-tDcp-1
c
anti-DEVdG - adult posterior midgut
anti-DEVdG
40 µm
intensity (arb. units)
distance (µm)
0
4
8
20
40

Supplementary Figure 4: Epimerisations make consensus cleavage sites resist proteolytic cleavage by caspases *in vivo*

a, Confocal images of dissected female posterior guts of homochiral (control) and heterochiral (*Pimt*-RNAi) imagoes. Animals express *UAS-CD8::PARP1::Venus* and *UASp-tDcp-1* under the control of *Act5C-Gal4*.

b, Quantification of the ratio of signal intensities. Each experiment was repeated independently at least three times, n=number of cells. Values are presented as average ± standard deviation (S.D.). P-value from two-sided Mann-Whitney U-test is ****p<0.0001.

c, Anti-DEV***d***G antibody was used to detect uncleaved DEV***d***G sequences in PARP1-expressing tissues. Fluorescent intensities of anti-DEV***d***G (red) and Venus (green) are measured by ImageJ software at the yellow lines. Each experiment was repeated independently at least three times. Source data are provided as a Source Data file for b, and c,.

Multiple sequence comparison of Pimt from different species

```
C.c.h.          ----------------------------------------MRLNQFFCTIFLIATFILIT
D.m.PA          -----------------------------------------------------------M
D.m.PC          -----------------------------------------------------------M
D.r.            ---------------------MSGDDVSVLEVVTVVGRAVCAGAALTAALYLIRRVCLIM
H.s.            -----------------------------------------------------------M
M.m.            MPGARIGGSGSDGSNSGRSSGDTSGAVTVWEVVSLLGKLLGTVAALKVVLYLL-RVCFAM


C.c.h.          TMIFNGLYEASQNKLVRNLSQRYPFKSQRTKEVMLLVDRADFTNEHPYADFPQQIGFGAT
D.m.PA          AWRSVG---ANNEDLIRQLKDHGVIASDAVAQAMKETDRKHYSPRNPYMDAPQPIGGGVT
D.m.PC          AWRSVG---ANNEDLIRQLKDHGVIASDAVAQAMKETDRKHYSPRNPYMDAPQPIGGGVT
D.r.            AWKSGG---ASHAELVNNLRKNGIIKSDRVYEVMLATDRSHFSRCNPYMDSPQSIGYQAT
H.s.            AWKSGG---ASHSELIHNLRKNGIIKTDKVFEVMLATDRSHYAKCNPYMDSPQSIGFQAT
M.m.            AWKSGG---ASHSELIHNLRKNGIIKTDKVFEVMLATDRSHYAKSNPYMDSPQSIGFQAT
                :    *   *.: .*:.:* ..  : :: . :.*  .**  ::  :** * ** **  .*

C.c.h.          ISAPHMHALAIDLLEPVINEDSHILDVGSGSGYLAVCFAKMVGSNG-----TVYGIDHIE
D.m.PA          ISAPHMHAFALEYLRDHLKPGARILDVGSGSGYLTACFYRYIKAKGVDADTRIVGIEHQA
D.m.PC          ISAPHMHAFALEYLRDHLKPGARILDVGSGSGYLTACFYRYIKAKGVDADTRIVGIEHQA
D.r.            ISAPHMHAYALELLHDHLYEGAKALDVGSGSGILSVCFSRMVGPTG-----KVIGIDHIK
H.s.            ISAPHMHAYALELLFDQLHEGAKALDVGSGSGILTACFARMVGCTG-----KVIGIDHIK
M.m.            ISAPHMHAYALELLFDQLHEGAKALDVGSGSGILTACFARMVGNSG-----KVIGIDHIK
                ******** *:: *   :  .:. ******** *:.** . :  .*      : **:*

C.c.h.          DLVNCSKQNIRKNNADLLDSKKLILILGDGRLGYPQGAPYDAIHVGAAPEEIPKALIDQL
D.m.PA          ELVRRSKANLNTDDRSMLDSGQLLIVEGDGRKGYPPNAPYNAIHVGAAAPDTPTELINQL
D.m.PC          ELVRRSKANLNTDDRSMLDSGQLLIVEGDGRKGYPPNAPYNAIHVGAAAPDTPTELINQL
D.r.            ELVEDSIANVKKDDPSLITSGRIKLIVGDGRMGFTEEAPYDAIHVGAAAPTVPQALLDQL
H.s.            ELVDDSVNNVRKDDPTLLSSGRVQLVVGDGRMGYAEEAPYDAIHVGAAAPVVPQALIDQL
M.m.            ELVDDSITNVKKDDPMLLSSGRVRLVVGDGRMGYAEEAPYDAIHVGAAAPVVPQALIDQL
                :**  *  *:..::  :: * .: :: **** *:.  ***:*******.   *  *::**

C.c.h.          AIGGRMVIPVGLAGEQQFL-QVDKISEKEVRKQVITAVNYVPLTDREQQLQL---
D.m.PA          ASGGRLIVPVGPDGGSQYMQQYDKDANGKVEMTRLMGVMYVPLTDLRS-------
D.m.PC          ASGGRLIVPVGPDGGSQYMQQVGRC------------------------------
D.r.            KPGGRLILPVGPAGGNQMLEQYDKLEDGSTKMKPLMGVIYVPLTDKDKQWSRWK-
H.s.            KPGGRLILPVGPAGGNQMLEQYDKLQDGSIKMKPLMGVIYVPLTDKEKQWSRDEL
M.m.            KPGGRLILPVGPAGGNQMLEQYDKLQDGSVKMKPLMGVIYVPLTDKEKQWSRDEL
                  ***:::***  * .* : * ..
```

Supplementary Figure 5: Pimt is evolutionary conserved.

Multiple sequence comparison of Pimt of various species. C.c.h.: *Candidatus Cardinium hertigii* (ROT47635.1) D.m.: *Drosophila melanogaster* PA and PC isoforms (NP_536756.1 and NP_001262287.1), D.r.: *Danio rerio* (XP_005160364.1), H.s.: *Homo sapiens* (NP_001347385.1), M.m.: *Mus musculus* (NP_001334157.1).

Supplementary Information on Molecular Modelling for

Title: Biological effects of the loss of homochirality in a multicellular organism

Model setup, molecular dynamics simulations and supplementary analyses

Model setup. The catalytic pocket of Caspase-3 houses the catalytic dyad residues Cys163 and His121 (see **Fig. 3d, Fig. S6a**) that facilitate the peptide bond cleavage of substrates with Asp-x-x-Asp sequences, such as the peptide bond between D and G in DEVDG peptide[1]. Mature Caspase-3 is formed by two active subunits, the larger P17 subunit spanning residues Asp29–Asp175 and the smaller P12 spanning Ser176–His277[2]. We used the 1.8 Å-resolution X-ray crystallographic structure of human Caspase-3 bound to the substrate *N*-acetyl-Asp-Glu-Val-Asp-chloromethyl ketone (Ac-DEVD-CMK) as our starting model (PDB code 2J30)[3]. Caspase-3 folds as a six-stranded central β-sheet surrounded by five α-helices with the substrate binding pocket active site flanked by four loops: L1 (residues 52–66, colored red in **Fig. S6a**), L2 (residues 163–175, green), L3 (residues 198–213, pink), and L4 (residues 247–263, cyan). The protease activation can be monitored by the recognition and cleavage of pentapeptide DEVDG[4], a consensus sequence in Poly(ADP-ribose) polymerase-1 (PARP1) in humans[5]. The reported human Caspase-3 PDB structure code 2J30 had N-terminal residues (Met1–Asp28) and residues Asp175–Asp179 missing, which we built in as loop regions using the *Modeller* code[6]. The pentapeptide DEV***D***G was constructed by removing the acetyl and chloromethyl ketone endgroups from Ac-DEVD-CMK and adding a peptide-bonded Gly amino acid at the carboxy end of Asp4’. The chiral point mutant DEV***d***G was then created from DEVDG (**Fig. S6b**) using the UCSF Chimera code[7]. **Fig. S6c** shows starting models of Caspase-3 bound with the native DEVDG ligand and with the chiral analogue DEV***d***G.

Simulation protocol. The Caspase-3/DEVDG and Caspase-3/DEV***d***G systems were sampled for a total of 0.36 microseconds of free molecular dynamics (MD). CHARMM36m forcefield parameters[8] were used to describe the DEVDG and DEV***d***G peptides and the Caspase-3 protein. CHARMM-modified TIP3P[9] was used to model the encompassing water with a minimum distance of 20 Å maintained between any protein atom and any edge of the simulation box. The MD simulations were carried out using the Gromacs 2018.4 code[10,11] with an integration time step of 2 fs implemented in the leapfrog integrator[12] with bond lengths to hydrogen constrained using the LINCS[13] (protein) and the SETTLE[14] (water) algorithms. Snapshots were saved every 2 ps. Background ions were added to neutralise protein formal charges and 150 mM NaCl was added to mimic physiological ionic strength. Periodic boundary conditions were applied to model bulk solvation with long-range electrostatics treated by the Particle Mesh Ewald (PME) method[15]. Protein and non-protein molecules (water and ions) were coupled separately to an external heat bath (310 K) with a coupling time constant of 1 ps using the velocity rescaling method[16]. All systems were energy minimised and then thermalised over 100 ps to 300K. The models were equilibrated for 1 ns in the constant-volume NVT ensemble followed by another 1 ns of NPT equilibration with the reference atmospheric pressure of 1 bar set using the Berendsen barostat[17]. The production runs were carried out for 180 ns for each complex in the constant-pressure NPT ensemble using the Parrinello-Rahman barostat[18].

Data processing, visualisation, and analysis. All analyses of peptide–Caspase-3 contact distances, hydrogen bonds, binding energies and secondary structure features (**Figs. S6e, S7a, b** below here) were performed using *Gromacs* tools and trajectories were visualised using the Visual Molecular Dynamics (VMD) program[19]. Hydrogen bond populations were computed by VMD using the standard cut-off distance of 0.35 nm and angle of 30 degrees. The fraction

of native contacts Q (**Fig. S6d**) in Caspase-3 was calculated using the definition from Best, Hummer and Eaton[20], implemented in the MDTraj[21] python library using the equation:

$$Q(X) = \frac{1}{N}\sum_{i,j}\frac{1}{1+\exp\left[\beta\left(r_{ij}(t)-\lambda r_{ij}^{0}\right)\right]} \quad \text{(S1)}$$

where N is the set of all pairs of heavy atoms (i, j), and heavy atoms i and j are in contact if the distance between them is less than 5 Å and they are separated by at least 3 residues. $r_{ij}(t)$ is the instantaneous distance between i and j in the structure sampled at time t and r_{ij}^{0} is the distance in the starting structure at time 0. β is a smoothening parameter taken to be 5 $Å^{-1}$ and λ is a factor that describes fluctuations when the contact is formed, taken to be 1.8 for the all-atom model. For more details on the method and choice of parameter values, please see ref.[20].

The binding free energies (ΔG) of pentapeptide DEV***D***G and its chiral point mutant DEV***d***G were computed from the molecular mechanics energies combined with Poisson-Boltzmann continuum solvation (MM/PBSA)[22] as implemented in Gromacs method *g_mmpbsa*[23]:

$$\Delta G = \Delta G_{bind,vacuum} + \Delta G_{solv,complex} - \left(\Delta G_{solv,ligand} + \Delta G_{solv,receptor}\right) \quad \text{(S2)}$$

$$\Delta G_{solv} = \Delta G_{polar} + \Delta G_{nonpolar} \quad \text{(S3)}$$

where $\Delta G_{bind,vacuum}$ = ΔG_{MM} (MM = molecular mechanics energy = electrostatic energy + vdW energy in vacuum), $\Delta G_{solv,complex}$ = free energy of solvation of the Caspase-3–DEV(D/***d***)G complex, $\Delta G_{solv,ligand}$ = solvation free energy of the peptide DEVDG or DEV***d***G, $\Delta G_{solv,receptor}$ = solvation free energy of Caspase-3, ΔG_{polar} = polar solvation free energy, *i.e.*, electrostatic component of solvation free energy, and $\Delta G_{nonpolar}$ = nonpolar solvation free energy obtained from a linear fit to the solvent accessible surface area (SASA)[23].

Supplementary analysis. The main findings from the simulations and their relation to the experiments are described in the main article. Here, further details are provided for the

interested reader. All simulation input and output files are available on request from the corresponding author.

To monitor the convergence of the simulations, we calculated timelines of the fraction of native contacts *Q(X)* of Caspase-3 (**Fig. S6d**). After the expected small <5% drop in *Q(X)* during the first ~0.1 microseconds relative to the constrained starting solid-state X-ray crystal structure, both the DEVDG and DEV***d***G bound complexes sample stable Caspase-3 conformational states for the remainder of dynamics, and so we use the last 80 ns to compute average properties and hydrogen bond (H-bond) populations. To assess the strength of H-bonding between Caspase-3 and the pentapeptides, we map timelines of H-bond counts (**Fig. S6e**) and percent existence (*i.e.*, occupancies) of H-bonds (**Supplementary Tables 1 and 2**). A steep decline in H-bond population occurs after around 0.14 microseconds of dynamics leading to a complete loss of H-bonds for the Caspase-3/DEV***d***G system. This rupture of the Caspase-3/DEV***d***G complex indicates that a steric misfit between the chiral D-Asp point mutant and the binding pocket residues ejects DEV***d***G from the Caspase-3 active site (**Fig. S6e**). By contrast, DEVDG forms a strong and stable network of H-bonds with the Caspase-3 binding pocket (**Tables S1 and S2**), highlighting the evolved chiral specificity of Caspase-3 for binding DEVDG and rejecting DEV***d***G.

To gain a deeper understanding of the detailed pentapeptide–Caspase-3 interactions that enforce the chiral specificity, we plotted the timelines of interaction energies (**Fig. S7a**). We observe that both DEVDG–Caspase-3 and DEV***d***G–Caspase-3 interactions are driven by electrostatics with DEV***d***G losing contacts with Caspase-3 due to Coulomb repulsion of the non-native heterochiral peptide. We note from the MD trajectories that DEVDG may form a β-sheet structure that complements a nearby sub-domain of loop L3 (residues Ser205–Gly212), as determined by computing the time evolution of secondary structures of the DEV(D/***d***)G peptide and the 205 –212 sub-domain in L3 during the first 140 ns of peptide-bound dynamics

for both ligands (**Fig. S7b**). For DEVDG/Caspase-3, we find that DEVDG and L3 show persistent β-sheets (with short spans of β-bridge) throughout the dynamics. By contrast, DEV***d***G loses its β-structure after just 5 ns of dynamics (**Fig. S7b**) and continues to sample just β-bridge like structure (*i.e.*, short fragments show β-sheet-like binding patterns) until it exits the pocket after ~140 ns of dynamics to fully uncoil in water. The timelines of binding energies (ΔG) show that binding of DEV***d***G to Caspase-3 becomes completely unfavourable (**Fig. S7c**), in stark contrast to the persistent strong favorable binding of DEVDG. The modelling results highlight the major contribution of electrostatic interactions (balanced peptide–pocket and solvation interactions) in driving DEVDG–Caspase-3 complexation (**Fig. S7c**, right panel). Further decomposition of the net ΔG values into their residue-wise contributions ($\Delta G_{contrib}$) reveals the role of positively charged Caspase-3 arginine residues Arg67 and Arg207 (**Fig. S7d**) in maintaining the strong H-bond network with DEVDG peptide Asp and Glu residues that enforces the homochiral specificity.

We further mapped the distance timelines of molecular recognition of DEVDG and contrasting repulsion of DEV***d***G to Caspase-3 residues, identifying residues with chiral discriminating capacity as expressed through their binding free energy components of $\Delta G_{contrib} < -10$ kJ/mol and $\Delta G_{contrib} > +10$ kJ/mol, respectively (**Fig. S8**). We observe that residues Arg64, Arg207, Lys210 and Ser249 facilitate binding of the N-terminus of the chiral mutant DEV***d***G in the catalytic pocket through favourable electrostatic contacts (**Fig. S8a**), despite competing repulsions from Asp70 and Asp211. After ~140 ns of free dynamics, these repulsions become stronger with additional repulsive contribution of Asp253 against the C-terminus of DEV***d***G, triggering release of the chiral analogue from the catalytic pocket (**Fig. S8b**). The timelines of these repulsive forces suggests that at ~140 ns, the first release trigger is initiated by residues Asp70 and 211, followed by repulsion from Asp253 at 150 ns, which makes DEV***d***G lose complete contact with Caspase-3. At ~163 ns, DEV***d***G hovers around the vicinity of Caspase-

3 before complete departure at 170 ns with the binding pocket closing and inhibiting further recognition of the chiral mutant (**Fig. S8b**).

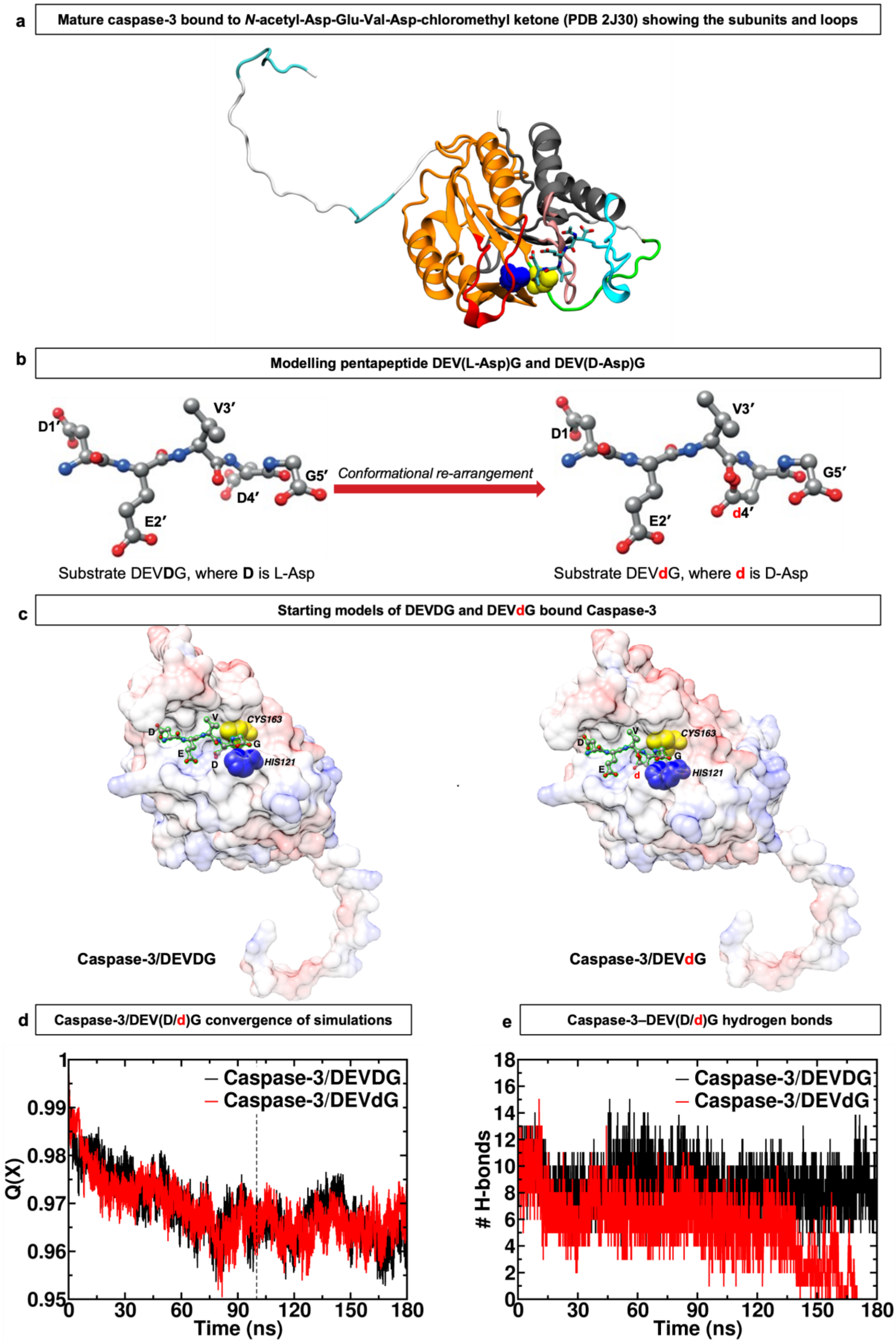
a
Mature caspase-3 bound to N-acetyl-Asp-Glu-Val-Asp-chloromethyl ketone (PDB 2J30) showing the subunits and loops
b
Modelling pentapeptide DEV(L-Asp)G and DEV(D-Asp)G
D1′
V3′
G5′
D4′
E2′
Conformational re-arrangement
D1
V3′
G5′
d4′
E2′
Substrate DEVDG, where D is L-Asp
Substrate DEVdG, where d is D-Asp
c
Starting models of DEVDG and DEVdG bound Caspase-3
CYS163
HIS121
D
V
E
G
D
d
Caspase-3/DEVDG
Caspase-3/DEVdG
d
Caspase-3/DEV(D/d)G convergence of simulations
Caspase-3/DEVDG
Caspase-3/DEVdG
Q(X)
Time (ns)
e
Caspase-3–DEV(D/d)G hydrogen bonds
Caspase-3/DEVDG
Caspase-3/DEVdG
H-bonds
Time (ns)

Supplementary Figure 6.

a, Structure of mature Caspase-3 bound to *N*-acetyl-Asp-Glu-Val-Asp-chloromethyl ketone (Ac-DEVD-CMK; PDB code 2J30[3]). Subunits P17 and P12 are shown in orange and grey cartoons, respectively. Loops L1, L2, L3 and L4 are shown in red, green, pink and cyan cartoons, respectively. The residues forming catalytic dyad His121–Cys163 are shown in blue and yellow spheres, respectively. The substrate Ac-DEVD-CMK is shown as sticks.

b, Modelled ball and stick structures of pentapeptide DEVDG and its stereoisomer DEVdG.

c, Initial models of DEVDG and DEVdG (shown as ball and stick) bound to Caspase-3 enzyme with the catalytic dyad His121 (blue) and Cys163 (yellow) highlighted and the protein overlaid with its electrostatic potential surface. Timelines of **d,** Caspase-3 fraction of native contacts Q(X) showing convergence of simulations after 100 ns of equilibrated dynamics, and **e,** number of hydrogen bonds (H-bonds) between pentapeptide and Caspase-3 for both Caspase-3/DEVDG and Caspase-3/DEVdG complexes.

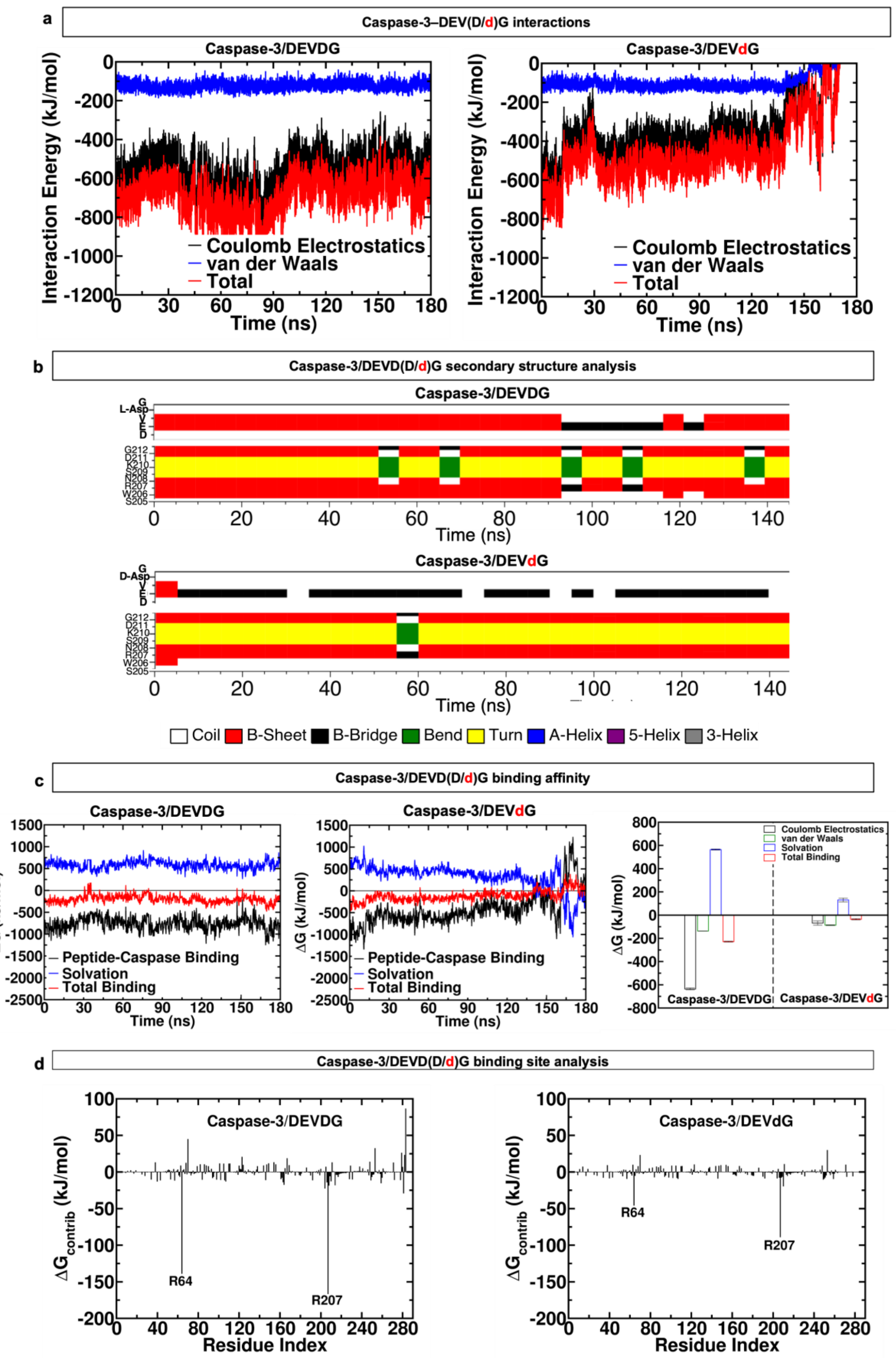
a
Caspase-3–DEV(D/d)G interactions
Caspase-3/DEVDG
Caspase-3/DEVdG
Interaction Energy (kJ/mol)
Time (ns)
Coulomb Electrostatics
van der Waals
Total
b
Caspase-3/DEVD(D/d)G secondary structure analysis
Caspase-3/DEVDG
Caspase-3/DEVdG
L-Asp
D-Asp
G212
D211
K210
S209
N208
R207
W206
S205
Time (ns)
Coil
B-Sheet
B-Bridge
Bend
Turn
A-Helix
5-Helix
3-Helix
c
Caspase-3/DEVD(D/d)G binding affinity
Caspase-3/DEVDG
Caspase-3/DEVdG
ΔG (kJ/mol)
Time (ns)
Peptide-Caspase Binding
Solvation
Total Binding
Coulomb Electrostatics
van der Waals
Solvation
Total Binding
d
Caspase-3/DEVD(D/d)G binding site analysis
Caspase-3/DEVDG
Caspase-3/DEVdG
ΔG contrib (kJ/mol)
R64
R207
Residue Index

Supplementary Figure 7.

a, Timelines of interaction energies between DEVD(D/d)G and Caspase-3.

b, Timelines of secondary structures of DEV(D/d)G and the sub-domain in loop L3 (residues 205–212) of Caspase-3.

c, Timelines of binding energies (ΔG) of DEV(D/d)G to Caspase-3, and bar plot of their average ΔG during the final 80 ns of dynamics. (n = 400 independent data points). The data is presented as mean values ± SEM. Error bars show estimate of the standard deviation of the sampling distribution of the mean. The average binding energy and the associated confidence interval was calculated by a bootstrap analysis. The autocorrelation function of binding energy values of simulation snapshots every 20 ps were obtained, which showed the snapshots are uncorrelated, and was a primary requirement for bootstrapping.

d, Residue-wise contribution to total binding energy ($\Delta G_{contrib}$).

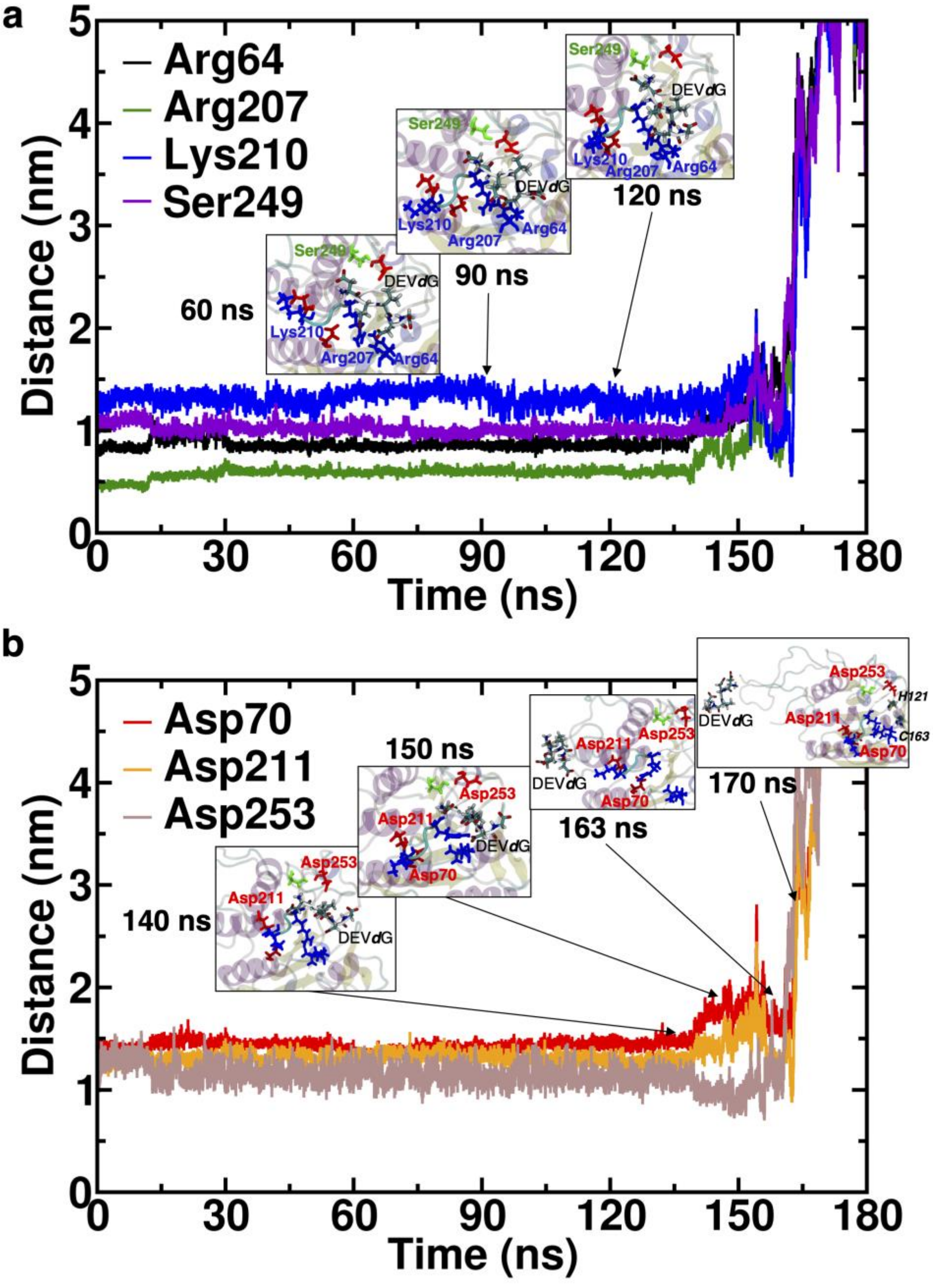

a
Arg64
Arg207
Lys210
Ser249
60 ns
90 ns
120 ns
Ser249
DEVdG
Lys210
Arg207
Arg64
Distance (nm)
Time (ns)
b
Asp70
Asp211
Asp253
140 ns
150 ns
163 ns
170 ns
Asp211
Asp253
Asp70
DEVdG
H121
C163
Distance (nm)
Time (ns)

Supplementary Figure 8.

a, Timelines of centres of mass (COM) distances between Caspase-3 residues (Arg64, Arg207, Lys210 and Ser249) that make favourable contacts during the first ~140 ns of dynamics with the DEVD***d***G chiral mutant.

b, Timelines of COM distances for Caspase-3 residues (Asp 70, 211 and 253) that repel DEVD***d***G, and ultimately trigger its release after ~140 ns of dynamics.

Supplementary Tables

Donor residue	**Acceptor residue**	**Occupancy (%)**
Arg207 (Caspase-3)	L-Asp4’ (DEVDG)	90.0
Glu2’ (DEVDG)	Arg207 (Caspase-3)	73.6
Arg64 (Caspase-3)	L-Asp4’ (DEVDG)	66.0
Arg207 (Caspase-3)	Glu2’ (DEVDG)	64.5
Asn208 (Caspase-3)	Asp1’ (DEVDG)	61.5
Trp214 (Caspase-3)	Asp1’ (DEVDG)	51.6
Phe250 (Caspase-3)	Asp1’ (DEVDG)	25.7
Asp1’ (DEVDG)	Phe250 (Caspase-3)	25.6
Arg207 (Caspase-3)	Glu2’ (DEVDG)	24.9
Thr62-side (Caspase-3)	Gly5’-side (DEVDG)	24.5
Thr204 (Caspase-3)	Gly5’ (DEVDG)	13.3
Thr62-side (Caspase-3)	Gly5’-main (DEVDG)	10.5

Supplementary Table 1. Hydrogen bond statistics between Caspase-3 and DEV***D***G

Donor residue	**Acceptor residue**	**Occupancy (%)**
Arg207 (Caspase-3)	D-Asp4’ (DEVdG)	37.4
Phe250 (Caspase-3)	Asp1’ (DEVdG)	29.7
Arg207 (Caspase-3)	Glu2’ (DEVdG)	24.1
Glu2’ (DEVdG)	Arg207 (Caspase-3)	24.0
Asn208 (Caspase-3)	Asp1’ (DEVdG)	19.6
Asp1’ (DEVdG)	Phe250 (Caspase-3)	18.3
Ser251 (Caspase-3)	Asp1’ (DEVdG)	11.3

Supplementary Table 2. Hydrogen bond statistics between Caspase-3 and DEV***d***G

**Figures**	Chr X.	Chr II.	Chr III.	Simplified on figure subtitles
**1b-c**	w^{1118}	+	*Pimt KO #1 / +*	control
	w^{1118}	+	*Pimt KO #1 / Pimt KO #2*	$Pimt^{n1}$
**4b-c**	*y, w*	*nub-Gal4 / UAS-LacZ*-RNAi	*UAS-CD8::PARP1::Venus* (DEVD), *UAS-tDcp1 / +*	control
	y, w	*nub-Gal4 / UAS-Pcmt-*RNAi^{GD19123}	*UAS-CD8::PARP1::Venus* (DEVD), *UAS-tDcp1 / +*	*Pimt*-RNAi
	y, w	*nub-Gal4 / UAS-LacZ*-RNAi	*UAS-CD8::PARP1::Venus* (DEVG), *UAS-tDcp1 / +*	control
	y, w	*nub-Gal4 / UAS-Pcmt-*RNAi^{GD19123}	*UAS-CD8::PARP1::Venus* (DEVG), *UAS-tDcp1 / +*	*Pimt*-RNAi
**4d**	*y, w*	*nub-Gal4 / UAS-LacZ*-RNAi	*UAS-CD8::PARP1* (DEVD)*::Venus, UAS-tDcp1 / +*	control
	y, w	*nub-Gal4 / UAS-Pcmt-*RNAi^{GD19123}	*UAS-CD8::PARP1* (DEVD)*::Venus, UAS-tDcp1 / +*	*Pimt*-RNAi
**5a**	w^{1118}	+	+	control
	w^{1118}	+	*Pimt KO #1 / Pimt KO #2*	$Pimt^{n1}$
	w^{1118}	+	*Pimt* KI^{WT} *#1 / Pimt Pimt* KI^{WT} *#2*	$Pimt^{WT}$
	w^{1118}	+	*Pimt* KI^{S60Q} *#1 / Pimt Pimt* KI^{60Q} *#2*	$Pimt^{S60Q}$
**5b**	*y, w, hsFlp*	*UAS-LacZ*-RNAi / +	*Act5C>CD2>Gal4, UAS-GFP / +*	control
	y, w, hsFlp	*UAS-Pcmt-*RNAi^{GD19123} / +	*Act5C>CD2>Gal4, UAS-GFP / +*	*Pimt*-RNAi
**5c**	*y, w, hsFlp*	*UAS-LacZ*-RNAi / *UAS-LacZ*-RNAi	*Act5C>CD2>Gal4, UAS-GFP / +*	control
	y, w, hsFlp	*UAS-Pcmt-*RNAi^{GD19123} / *UAS-LacZ*-RNAi	*Act5C>CD2>Gal4, UAS-GFP / +*	*Pimt*-RNAi
	y, w, hsFlp / UAS-$Pimt^{BDSC}$ 27394	*UAS-Pcmt-*RNAi^{GD19123} / +	*Act5C>CD2>Gal4, UAS-GFP / +*	*Pimt*-RNAi, *UAS-Pimt (I)*
	y, w, hsFlp	*Pcmt*-RNAi^{GD19123} / *UAS-*$Pimt^{BDSC27393}$	*Act5C>CD2>Gal4, UAS-GFP / +*	*Pimt*-RNAi, *UAS-Pimt (II)*
	y, w, hsFlp	*UAS-Pcmt-*RNAi^{GD19123}/ +	*Act5C>CD2>Gal4, UAS-GFP / UAS-*$Pimt^{BDSC27396}$	*Pimt*-RNAi, *UAS-Pimt (III)*
**6a,c,e**	*y, w*	*esg-*$Gal4^{NP7397}$*, UAS-GFP, Tub-*$Gal80^{TS}$ */ UAS-LacZ*-RNAi	*UAS-Notch*-RNAi /	esg^{TS}*>GFP, Notch*-RNAi + control
**6b,d,e**	*y, w*	*esg-*$Gal4^{NP7397}$*, UAS-GFP, Tub-*$Gal80^{TS}$ */ UAS-Pimt-*RNAi^{GD19123}	*UAS-Notch*-RNAi / +	esg^{TS}*>GFP, Notch*-RNAi + *Pimt-*RNAi
**6f,g**	*y, w*	*esg-*$Gal4^{NP7397}$*, UAS-GFP, Tub-*$Gal80^{TS}$ */ UAS-LacZ*-RNAi	*UAS-Notch*-RNAi / +	esg^{TS}*>GFP, Notch*-RNAi + control
	y, w	*esg-*$Gal4^{NP7397}$*, UAS-GFP, Tub-*$Gal80^{TS}$ */ UAS-Pimt-*RNAi^{GD19123}	*UAS-Notch*-RNAi / +	esg^{TS}*>GFP, Notch*-RNAi + *Pimt-*RNAi
	y, w	*esg-*$Gal4^{NP7397}$*, UAS-GFP, Tub-*$Gal80^{TS}$ */ UAS-Pimt-*RNAi^{GD19123}	*UAS-Notch*-RNAi / *UAS-*$Pimt^{wt}$*-HA*	esg^{TS}*>GFP, Notch*-RNAi + *Pimt-*RNAi, *UAS-Pimt*
**Supplementary Figures**				

**S1b**	w^{1118}	*UAS-Stinger(GFP)*^{NLS} / +	*Pimt-Gal4 / +*	
**S2a-b**	*y, w*	*Act5C-Gal4* / *UAS-LacZ*-RNAi	+ / +	*Act-Gal4*>control
	y, w	*Act5C-Gal4* / *UAS-Pimt*-RNAi^{GD19123}	+ / +	*Act-Gal4>Pimt*-RNAi
	w^{1118}	+ / +	Pimt KO #4 / #5	$Pimt^{n1}$
**S2c-d**	w^{1118}	+	+	control
	w^{1118}	+	*Pimt KO #1 / Pimt KO #2*	$Pimt^{n1}$
	w^{1118}	+	*Pimt KO #3 / Pimt KO #4*	$Pimt^{n1}$
	w^{1118}	+	*Pimt KO #1 / Pimt KO #7*	$Pimt^{n1}$
**S4a-c**	*y, w*	*Act5C-Gal4 / UAS-LacZ*-RNAi	*UAS-CD8::PARP1::Venus* (DEVD), *UAS-tDcp1 / +*	*CD8::PARP1::Venus, UAS-tDcp1* + control
	y, w	*Act5C-Gal4 / UAS-Pcmt-*RNAi^{GD19123}	*UAS-CD8::PARP1::Venus* (DEVD), *UAS-tDcp1 / +*	*CD8::PARP1::Venus, UAS-tDcp1* + *Pimt*-RNAi

Supplementary Table 3: List of genotypes used in the study.

REAGENT or RESOURCE	SOURCE	IDENTIFIER
**Antibodies**		
Rabbit polyclonal anti-DEV***D***G	This study	H63-TAE
Rabbit polyclonal anti-DEV***βD***G	This study	pAb95-TAE
Rabbit polyclonal anti-DEV***d***G	This study	H65-TAE
Rabbit polyclonal anti-DEV***βd***G	This study	L23-TAE
**Synthetic oligopeptides**		
[C]-GG-DEV-[L-aspartate]-G-amide	This study	p41749
[C]-GG-DEV-[iso-L-aspartate]-G-amide	This study	p41747
[C]-GG-DEV-[D-aspartate]-G-amide	This study	p41748
[C]-GG-DEV-[iso-D-aspartate]-G-amide	This study	p41743
acetyl-DEVD-7-amido-4-methylcoumarin	This study	U8259FK120_5
acetyl-DEV-[iso-L-aspartate]-7-amido-4-methylcoumarin	This study	U8259FK120_9
acetyl-DEV-[D-aspartate]-7-amido-4-methylcoumarin	This study	U8259FK120_7
acetyl-[iso-D-aspartate]-7-amido-4-methylcoumarin	This study	U8259FK120_11
acetyl-DEVG-7-amido-4-methylcoumarin	This study	U8259FK120_15
acetyl-DEVA-7-amido-4-methylcoumarin	This study	U8259FK120_13
acetyl-[D-aspartate]-[D-glutamate]-[D-valine]-[D-aspartate]-7-amido-4-methylcoumarin	This study	U8259FK120_17
acetyl-GGGGG-DEV-[L-aspartate]-GGGGGG-amide	This study	U6507FK170_1
acetyl-GGGGG-DEV-[iso-L-aspartate]-GGGGGG-amide	This study	U6507FK170_3
acetyl-GGGGG-DEV-[D-aspartate]-GGGGGG-amide	This study	U6507FK170_5
acetyl-KRKGDEVDGVDEVAK-amide	This study	U300BFJ200-1
acetyl-KRKGDEV-[iso-L-aspartate]-GVDEVAK-amide	This study	U300BFJ200-3
**Transgenic lines and mutants**		
$Pimt^{null1}$ mutant	This study	n.a.
$Pimt^{wt}$	This study	n.a.
$Pimt^{KI\ S60Q}$	This study	n.a.
Pimt-Gal4	This study	n.a.

Supplementary Table 4: Reagents or resources generated for this study.

Abbreviations:

AA: amino acid
Asp: aspartate
L-α-amino acid: L-amino acid
L-β-amino acid: iso-L-amino acid – establishes peptide bond via its β-carboxyl group
D-α-amino acid: D-amino acid
D-β-amino acid: iso-D-amino acid – establishes peptide bond via its β-carboxyl group

Supplementary References

1 Poreba, M., Strozyk, A., Salvesen, G. S. & Drag, M. Caspase substrates and inhibitors. *Cold Spring Harb Perspect Biol* **5**, a008680, doi:10.1101/cshperspect.a008680 (2013).
2 Han, Z., Hendrickson, E. A., Bremner, T. A. & Wyche, J. H. A sequential two-step mechanism for the production of the mature p17:p12 form of caspase-3 in vitro. *J Biol Chem* **272**, 13432-13436, doi:10.1074/jbc.272.20.13432 (1997).
3 Feeney, B., Pop, C., Swartz, P., Mattos, C. & Clark, A. C. Role of loop bundle hydrogen bonds in the maturation and activity of (Pro)caspase-3. *Biochemistry* **45**, 13249-13263, doi:10.1021/bi0611964 (2006).
4 Zhang, J. *et al.* Visualization of caspase-3-like activity in cells using a genetically encoded fluorescent biosensor activated by protein cleavage. *Nat Commun* **4**, 2157, doi:10.1038/ncomms3157 (2013).
5 Nicholson, D. W. *et al.* Identification and inhibition of the ICE/CED-3 protease necessary for mammalian apoptosis. *Nature* **376**, 37-43, doi:10.1038/376037a0 (1995).
6 Eswar, N. *et al.* Comparative protein structure modeling using Modeller. *Current protocols in bioinformatics* **15**, 5.6. 1-5.6. 30 (2006).
7 Pettersen, E. F. *et al.* UCSF Chimera--a visualization system for exploratory research and analysis. *J Comput Chem* **25**, 1605-1612, doi:10.1002/jcc.20084 (2004).
8 Huang, J. *et al.* CHARMM36m: an improved force field for folded and intrinsically disordered proteins. *Nat Methods* **14**, 71-73, doi:10.1038/nmeth.4067 (2017).
9 MacKerell, A. D. *et al.* All-atom empirical potential for molecular modeling and dynamics studies of proteins. *J Phys Chem B* **102**, 3586-3616, doi:10.1021/jp973084f (1998).
10 Berendsen, H. J., van der Spoel, D. & van Drunen, R. GROMACS: a message-passing parallel molecular dynamics implementation. *Computer physics communications* **91**, 43-56 (1995).
11 Van Der Spoel, D. *et al.* GROMACS: fast, flexible, and free. *J Comput Chem* **26**, 1701-1718, doi:10.1002/jcc.20291 (2005).
12 Hockney, R. W. The potential calculation and some applications. *Methods Comput. Phys.* **9**, 136 (1970).
13 Hess, B., Bekker, H., Berendsen, H. J. C. & Fraaije, J. G. E. M. LINCS: A linear constraint solver for molecular simulations. *Journal of Computational Chemistry* **18**, 1463-1472, doi:doi:10.1002/(SICI)1096-987X(199709)18:12<1463::AID-JCC4>3.0.CO;2-H (1997).

14 Miyamoto, S. & Kollman, P. A. Settle: An analytical version of the SHAKE and RATTLE algorithm for rigid water models. *Journal of Computational Chemistry* **13**, 952-962, doi:doi:10.1002/jcc.540130805 (1992).

15 Darden, T., York, D. & Pedersen, L. Particle mesh Ewald: An N· log (N) method for Ewald sums in large systems. *The Journal of chemical physics* **98**, 10089-10092 (1993).

16 Bussi, G., Donadio, D. & Parrinello, M. Canonical sampling through velocity rescaling. *J Chem Phys* **126**, 014101, doi:10.1063/1.2408420 (2007).

17 Berendsen, H. J., Postma, J. v., van Gunsteren, W. F., DiNola, A. & Haak, J. Molecular dynamics with coupling to an external bath. *The Journal of chemical physics* **81**, 3684-3690 (1984).

18 Parrinello, M. & Rahman, A. Polymorphic transitions in single crystals: A new molecular dynamics method. *Journal of Applied Physics* **52**, 7182-7190, doi:10.1063/1.328693 (1981).

19 Humphrey, W., Dalke, A. & Schulten, K. VMD: visual molecular dynamics. *J Mol Graph* **14**, 33-38 (1996).

20 Best, R. B., Hummer, G. & Eaton, W. A. Native contacts determine protein folding mechanisms in atomistic simulations. *Proc Natl Acad Sci U S A* **110**, 17874-17879, doi:10.1073/pnas.1311599110 (2013).

21 McGibbon, R. T. *et al.* MDTraj: A Modern Open Library for the Analysis of Molecular Dynamics Trajectories. *Biophys J* **109**, 1528-1532, doi:10.1016/j.bpj.2015.08.015 (2015).

22 Xu, L., Sun, H., Li, Y., Wang, J. & Hou, T. Assessing the performance of MM/PBSA and MM/GBSA methods. 3. The impact of force fields and ligand charge models. *J Phys Chem B* **117**, 8408-8421, doi:10.1021/jp404160y (2013).

23 Kumari, R., Kumar, R., Open Source Drug Discovery, C. & Lynn, A. g_mmpbsa--a GROMACS tool for high-throughput MM-PBSA calculations. *J Chem Inf Model* **54**, 1951-1962, doi:10.1021/ci500020m (2014).
