# Supplementary material for: Biological effects of the loss of homochirality in a multicellular organism
Source: Nat Commun. 2022 Nov 18;13:7059. doi: 10.1038/s41467-022-34516-x (PMC9674851; doi:10.1038/s41467-022-34516-x)
Supplement: Supplementary file 3 — Reporting Summary [file 41467_2022_34516_MOESM3_ESM.pdf]

nature portfolio

Corresponding author(s): Agnes Banreti

Last updated by author(s): 17/10/2022

Reporting Summary

Nature Portfolio wishes to improve the reproducibility of the work that we publish. This form provides structure for consistency and transparency in reporting. For further information on Nature Portfolio policies, see our Editorial Policies and the Editorial Policy Checklist.

Statistics

For all statistical analyses, confirm that the following items are present in the figure legend, table legend, main text, or Methods section.

n/a	Confirmed	
☐	☒	The exact sample size (n) for each experimental group/condition, given as a discrete number and unit of measurement
☐	☒	A statement on whether measurements were taken from distinct samples or whether the same sample was measured repeatedly
☐	☒	The statistical test(s) used AND whether they are one- or two-sided<br>*Only common tests should be described solely by name; describe more complex techniques in the Methods section.*
☒	☐	A description of all covariates tested
☒	☐	A description of any assumptions or corrections, such as tests of normality and adjustment for multiple comparisons
☐	☒	A full description of the statistical parameters including central tendency (e.g. means) or other basic estimates (e.g. regression coefficient) AND variation (e.g. standard deviation) or associated estimates of uncertainty (e.g. confidence intervals)
☐	☒	For null hypothesis testing, the test statistic (e.g. F, t, r) with confidence intervals, effect sizes, degrees of freedom and P value noted<br>*Give P values as exact values whenever suitable.*
☒	☐	For Bayesian analysis, information on the choice of priors and Markov chain Monte Carlo settings
☒	☐	For hierarchical and complex designs, identification of the appropriate level for tests and full reporting of outcomes
☐	☒	Estimates of effect sizes (e.g. Cohen's d, Pearson's r), indicating how they were calculated

Our web collection on statistics for biologists contains articles on many of the points above.

Software and code

Policy information about availability of computer code

Data collection	For confocal images, ZEISS ZEN software was used for data collection/capturing images.<br>For SRCD measurements, CDtool software was used for data collection.
Data analysis	All statistical analyses were carried out using ImageJ (2015), Microsoft Excel (version 16.16.27), CDtool and GraphPad Prism 9.2.0 (283).

For manuscripts utilizing custom algorithms or software that are central to the research but not yet described in published literature, software must be made available to editors and reviewers. We strongly encourage code deposition in a community repository (e.g. GitHub). See the Nature Portfolio guidelines for submitting code & software for further information.

Data

Policy information about availability of data

All manuscripts must include a data availability statement. This statement should provide the following information, where applicable:

- Accession codes, unique identifiers, or web links for publicly available datasets
- A description of any restrictions on data availability
- For clinical datasets or third party data, please ensure that the statement adheres to our policy

All data is available in the main text or the supplementary data. Source data are provided with this paper. Materials generated for the study are available from the corresponding author on reasonable request.

Field-specific reporting

Please select the one below that is the best fit for your research. If you are not sure, read the appropriate sections before making your selection.

☒ Life sciences ☐ Behavioural & social sciences ☐ Ecological, evolutionary & environmental sciences

For a reference copy of the document with all sections, see nature.com/documents/nr-reporting-summary-flat.pdf

Life sciences study design

All studies must disclose on these points even when the disclosure is negative.

Sample size	No a priori sample-size calculation was performed, sample size was set according to the reproducibility of each experiment. ARRIVE guidelines had been followed for the study and the maximum number of replicates were used for each experiment above which additional replicates did not alter the statistical significance.
Data exclusions	No data were excluded from the analyses.
Replication	Each unique experiments were repeated at least three independent times (N). "n" refers to the number of biological replicates for each experimental groups. At least 9 biological replicates per genotype and/or condition were analysed. The number of technical replicates, the experimental units, and number of experimental units allocated to each group are indicated for all experiments in the Figures and/or in the Figure legends. All attempts at replication were succesful.
Randomization	Samples were randomly selected for analysis and were randomly allocated into experimental groups.
Blinding	All experiments were conducted single-blind. Each experimental group were given numbers prior to dissection and analysis. Only after data were recorded experimental numbers were brought with the genotypes/treatment groups.

Reporting for specific materials, systems and methods

We require information from authors about some types of materials, experimental systems and methods used in many studies. Here, indicate whether each material, system or method listed is relevant to your study. If you are not sure if a list item applies to your research, read the appropriate section before selecting a response.

Materials & experimental systems

n/a	Involved in the study
☐	☒ Antibodies
☒	☐ Eukaryotic cell lines
☒	☐ Palaeontology and archaeology
☐	☒ Animals and other organisms
☒	☐ Human research participants
☒	☐ Clinical data
☒	☐ Dual use research of concern

Methods

n/a	Involved in the study
☒	☐ ChIP-seq
☒	☐ Flow cytometry
☒	☐ MRI-based neuroimaging

Antibodies

Antibodies used	Primary antibodies: Chicken anti-GFP (1:2000, ab13790, Abcam), Rabbit anti-Phospho-Histone H3 Ser10 (1:500, 9701L, Cell Signalling Technology), Rabbit anti-[C]-GG-DEV-[L-aspartate]-G-amide or anti-DEVDG (1:200, this work), Rabbit anti-[C]-GG-DEV-{iso-L-aspartate}-G-amide or DEVisoDG (1:200, this work), Rabbit anti-[C]-GG-DEV-{D-aspartate}-G-amide or DEVdG (1:200, this work), and Rabbit anti-[C]-GG-DEV-{iso-D-aspartate}-G-amide or DEVisodG (1:200, this work), Rabbit anti-α-Tubulin (1:1000, 2144S, Cell Signalling Technology).<br>Fluorescent secondary antibodies: Donkey anti-Rabbit Alexa Fluor 594 (Code: 711-585-152), Donkey anti-Rabbit Alexa Fluor 488 (Code: 711-546-152), Donkey anti-Chicken Alexa Fluor 488 (Code: 703-545-155) (all 1:2000, Jackson ImmunoResearch) and Goat anti-Mouse Alexa Fluor 647 (1:2000, A32728, Invitrogen).<br>HRP-conjugated secondary antibody: Donkey anti-Rabbit-HRP (1:2000, Amersham NA934-1ML).
Validation	Chicken anti-GFP (1:2000, ab13790, Abcam, validation: Abcam, Reacts with-Species independent)<br>Rabbit anti-phospho-histone H3 Ser10 (9701L, Cell Signalling Technology, validation: Cell Signalling Technology for W, IHC-P, IF-IC, Reacts with-D. melanogaster)<br>Rabbit anti-[C]-GG-DEV-[L-aspartate]-G-amide or anti-DEVDG (1:200, validation: in this study, Figures 1-2.)<br>Rabbit anti-[C]-GG-DEV-{iso-L-aspartate}-G-amide or DEVisoDG (1:200, validation: in this study, Figures 1-2.)<br>Rabbit anti-[C]-GG-DEV-{D-aspartate}-G-amide or DEVdG (1:200, validation: in this study, Figures 1-2.)<br>Rabbit anti-[C]-GG-DEV-{iso-D-aspartate}-G-amide or DEVisodG (1:200, validation: in this study, Figures 1-2.)<br>Rabbit anti-α-Tubulin (1:1000, 2144S, Cell Signalling Technology, validation: Cell Signalling Technology, highly specific and rigorously validated for WB, IHC, IF, F)

Animals and other organisms

Policy information about studies involving animals; ARRIVE guidelines recommended for reporting animal research

Laboratory animals	Drosophila melanogaster strains: w1118 isogenic strain (from B. Hudry), UAS-LacZ-RNAi (2nd, from M. Miura, w*;P{UAS-Pimt.C}2-1 (2nd, Bloomington Drosophila Stock Centre, BDSC: 27393), P{UAS-Pimt.C}X-2,w* (1st, BDSC: 27394), w*;P{UAS-Pimt.C}3-2 (3rd, BDSC: 27395), w*;P{UAS-Pimt.IR}3-4 (3rd, BDSC: 27396), P{UAS-Pimt.IR}X-5 (1st, BDSC: 27397), Pimtnull1 (3rd, this study, #1-#8, 8 lines in total), Pimt KI wt: Pimtwt (3rd, this study), Pimt KI S60Q: PimtS60Q (3rd, this study), UAS-Pimtwt-HA (3rd, this study), Pimt-Gal4 (3rd, this study), Cremini w+ (3rd, BDSC: 1501), ActGFP, Ser mini w+ (3rd, BDSC: 4534), w1118;UAS-StingerNLS (2nd, BDSC: 84277), nub-Gal4 (2nd, BDSC: 25754), Act5C-Gal4 (2nd, BDSC: 4414). UAS-Pimt-RNAi (2nd, Vienna Drosophila Resource Center, VDRC: GD19123), UAS-Pimt-RNAi (2nd, VDRC: GD19121), UAS-Notch-RNAi (3rd, VDRC: {GD14477}v27229), esg-Gal4NP7397, UAS-GFP, Tub-Gal80TS (2nd, from J. de Navascués), UAS-CD8::PARP1 (DEVD) and UAS-CD8::PARP1 (DEVG) (3rd, from Eli Arama); UAS-tDcp1 (deltaN-Dcp-1) (3rd, from Kim McCall).
Wild animals	No wild animals were used in the study.
Field-collected samples	No field collected samples were used in the study.
Ethics oversight	The production of rabbit antisera had been outsourced and carried out by Cambridge Research Biochemicals Limited, UK with Ethical approval.

Note that full information on the approval of the study protocol must also be provided in the manuscript.
